# Electron-Induced Perpendicular Graphene Sheets Embedded Porous Carbon Film for Flexible Touch Sensors

**DOI:** 10.1007/s40820-020-00480-8

**Published:** 2020-06-25

**Authors:** Sicheng Chen, Yunfei Wang, Lei Yang, Fouad Karouta, Kun Sun

**Affiliations:** 1 Key Laboratory of Education Ministry for Modern Design and Rotor-Bearing System, Xi’an Jiaotong University, Xi’an, 710049 People’s Republic of China; 2 Research School of Physics, The Australian National University, Canberra, ACT 2601 Australia

**Keywords:** Electron-induced perpendicular graphene, Porous nanostructure, Dual parameter, Flexible capacitance

## Abstract

Graphene-based materials on wearable electronics and bendable displays have received considerable attention for the mechanical flexibility, superior electrical conductivity, and high surface area, which are proved to be one of the most promising candidates of stretching and wearable sensors. However, polarized electric charges need to overcome the barrier of graphene sheets to cross over flakes to penetrate into the electrode, as the graphene planes are usually parallel to the electrode surface. By introducing electron-induced perpendicular graphene (EIPG) electrodes incorporated with a stretchable dielectric layer, a flexible and stretchable touch sensor with “in-sheet-charges-transportation” is developed to lower the resistance of carrier movement. The electrode was fabricated with porous nanostructured architecture design to enable wider variety of dielectric constants of only 50-μm-thick Ecoflex layer, leading to fast response time of only 66 ms, as well as high sensitivities of 0.13 kPa^−1^ below 0.1 kPa and 4.41 MPa^−1^ above 10 kPa, respectively. Moreover, the capacitance-decrease phenomenon of capacitive sensor is explored to exhibit an object recognition function in one pixel without any other integrated sensor. This not only suggests promising applications of the EIPG electrode in flexible touch sensors but also provides a strategy for internet of things security functions.

**Electronic supplementary material:**

The online version of this article (10.1007/s40820-020-00480-8) contains supplementary material, which is available to authorized users.

## Highlights

- An efficient method was proposed to prepare perpendicular graphene nano-sheets in flexible sensor electrode.
- The nanostructure in carbon electrode was fabricated to further enhance the sensitivity of sensor device using a simple method.
- The capacitance showed high performance within only 50-μm dielectric thickness, and an exciting phenomenon of decreasing in capacitance was analyzed.

**Electronic supplementary material:**

The online version of this article (10.1007/s40820-020-00480-8) contains supplementary material, which is available to authorized users.

## Graphical abstract

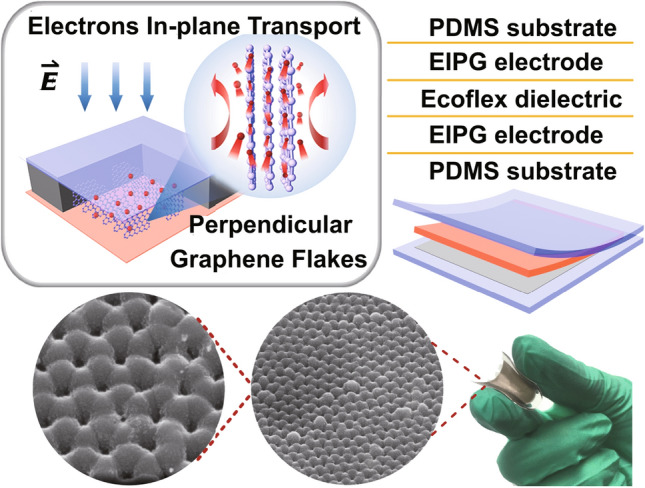

## Introduction

Graphene is one of the promising materials for flexible electronic devices as it can offer numerous benefits [1–3]. Hence, graphene-based materials are potential to be used within flexible sensors [4–6], including all transparent graphene e-skin [7], piezopotential powered graphene strain sensor [8], direct-contact tribotronic graphene device [9], and graphene sensory synapse [10]. The sensors can even operate under highly deformed states [11], such as folding [12, 13], twisting, and stretching [14]. Graphene-based capacitive touch sensor basically detects the applied pressure by transducing dielectric thickness variables between neighboring electrodes into electronic signals [15–18]. This implies that the polarized electric charges need to overcome the barrier of graphene sheets to cross over flakes to be collected by the electrode, which reduces the efficient utilization of large surface area in graphene layers [19, 20]. Although various graphene-based sensors have been demonstrated recently, whereby graphene-based materials have been fabricated as electrodes after spin coating or vacuum filtration procedures to form a continuous surface, polarized charges are still obstructed in most architectural design. Thus, in order to reply the ongoing challenges for graphene-based devices, more rational structures of perpendicular-graphene-based materials and architecture design are strongly required.

More importantly, micro-/nano-structure in the elastomeric electrode is thought to be a key element of sensor devices [21–24], providing faster response time, and higher sensitivity compared to the unstructured electrode [25, 26]. Therefore, structured graphene-based sensors have potential to possess superior performance [27]. Nevertheless, even the efficient fabrication of graphene-based materials on flexible electrode still remains a challenge, where fabricating graphene-composites can be time-consuming and costly and require specialized equipment [28], indicating a higher difficulty level on the architecture design of porous graphene-based electrode structure [29]. Recently, perpendicular graphene flakes embedded in carbon film show great advantages in a convenient fabrication process and excellent electrical properties [30]. Micro-/nano-structure combing with electron-induced perpendicular graphene (EIPG) is prospective in the field of graphene-based devices.

Inspired by the excellent electrical properties of EIPG materials, we demonstrate a highly sensitive (less graphene-sheets-barrier) wearable touch sensor by introducing the EIPG layer on the electrode, which presents effectively sensing capability to external pressure and object recognition at the same time. Enabled by the perpendicular orientation of graphene sheets embedded in carbon film fabricated in electron cyclotron resonance (ECR) sputtering system and porous structures in EIPG electrode, more accessible and efficient electron transport channels are realized [31], leading to fast and sensitive response upon quite wide pressure range. In addition, recognition of adjacent bio/non-bio objects can be realized according to the contact-short-circuit current ranging from 20 to 300 nA, together with the high sensitivity and flexibility of EIPG touch sensors, allows for the realization of adaption for various perceptions, thus suggesting potential applications in internet of things security functions.

## Experimental

### Synthesis of EIPG

The details of ECR sputtering system are mentioned in our former research [30], as shown in Fig. S1. The silicon substrate with 0.5 mm thickness was cut into squares with size of 20 × 20 mm^2^ and cleaned in acetone and ethanol bath by ultrasonic wave. Then, the sample was fixed onto the substrate holder followed by the evacuation of chamber. The working pressure would be 4.00 × 10^−2^ Pa after flowing argon gas. Argon plasma generated by microwave of 2.45 GHz with the power of 200 W was introduced to the chamber and confined between the magnetic mirrors set by a pair of coils. The magnetic coils current of 420 A was applied and formed a magnetic-mirror at both ends of the two coils with 390 mm distance. Then, electron irradiation was followed by applying a positive bias voltage on the sample substrate. To depositing EIPG electrode after generating argon plasma, the carbon target was connected to a bias of − 300 V and attracted argon ions. The carbon film was 200 nm thick after depositing for 40 min where a bias of + 100 V was applied to the sample substrate.

### Characterization

Surface morphology of AAO substrate and porous EIPG layer and cross-sectional structure of device were characterized by field-emission scanning electron microscope GeminiSEM 500 (ZEISS). Raman spectroscopy tests (Raman) were performed at laser Raman spectrometer. The graphene sheets crystallite was tested by field-emission transmission electron microscope (JEOL JEM-F200). The capacitance was measured by an Agilent E4980A Precision LCR meter, and short-circuit was collected by software platform written in LabVIEW connecting to a low-noise current preamplifier (SR570, Stanford Research System).

### Calculating Graphene Sheets Size

The graphene nanocrystallite size was analyzed based on the Raman results combing with the equation of *I*_*D*_*/I*_*G*_ = *C*(*λ*)*L*_*α*_^2^, Where the coefficient *C*(*λ*) is closely related to excitation laser wave length. In this research, the wavelength was set to be 532 nm with *C*(*λ*) of 0.55 nm^−2^, therefore calculating the graphene nanocrystallite size of the EIPG electrode layer (*I*_*D*_*/I*_*G*_ = 1.89) to be 1.85 nm.

## Results and Discussion

### Fabrication and Characterization of Device

**Fig. 1:**
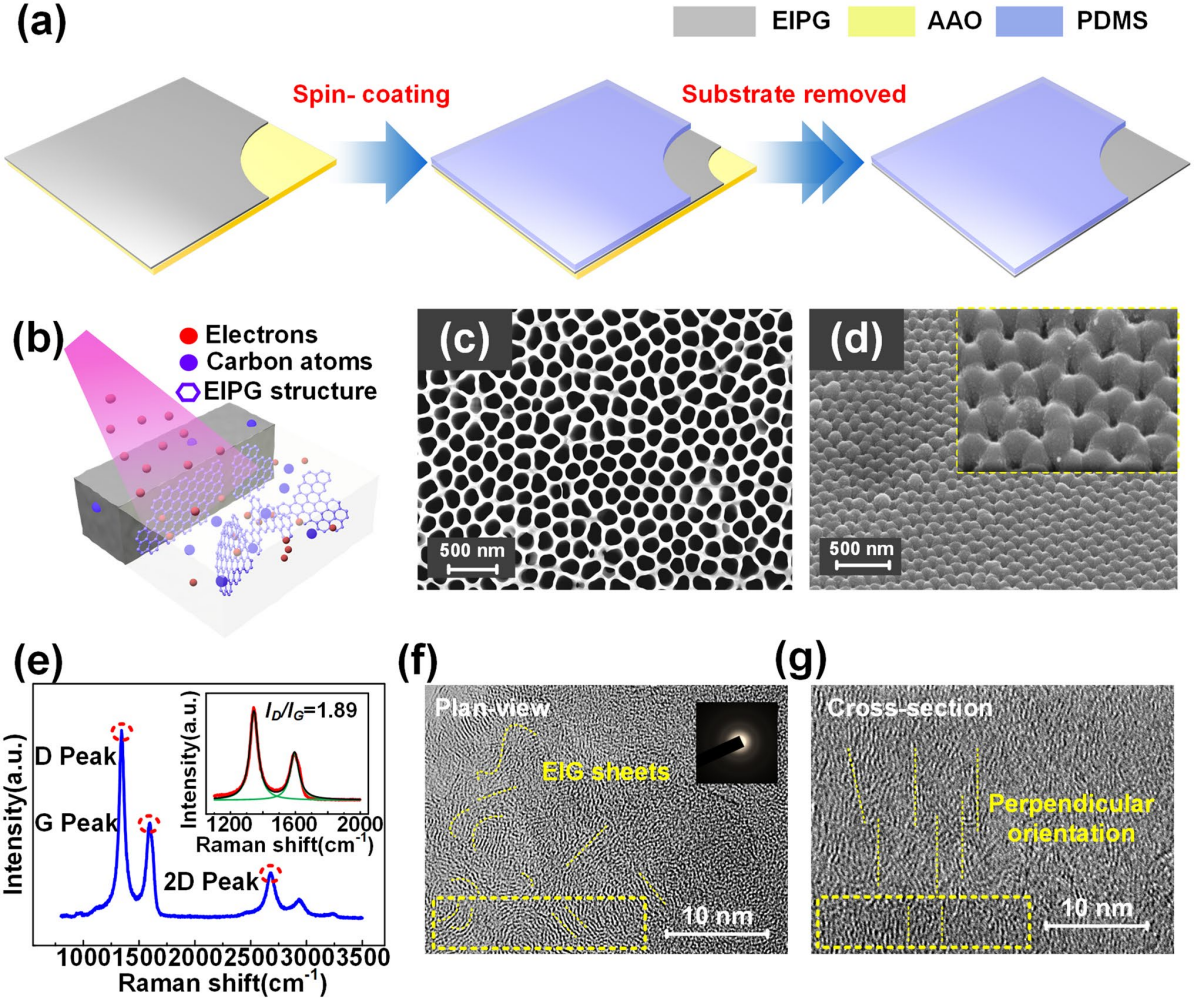
**a** Schematic illustration for the flexible EIPG electrode using spin coating and lift-off methodology. **b** Synthesizes process of perpendicular orientation of graphene sheets. **c** Field-emission scanning electron micrographs of AAO substrate and **d** deposited porous EIPG carbon film. **e** Raman spectrum of deposited EIPG electrode. **f** The HRTEM image of the plan-view of the EIPG electrode and **g** cross section of the EIPG electrode

The fabrication process for the flexible EIPG electrode is presented in Fig. 1a, where the conductive layer was directly deposited on the substrate, followed by spin coating of polydimethylsiloxane (PDMS), and the flexible electrode was obtained after removing the substrate. The thickness of conductive EIPG layer was 200 nm after depositing for 40 min (Fig. S2). Figure 1b shows the schematic illustration of graphene sheets structure induced by electrons irradiation during the depositing process of EIPG layer in ECR sputtering system (Fig. S1). Generally, porous structures on the electrode enable a more sensitive response in flexible piezocapacitive sensor devices [32, 33]. When an external stimulus is loaded, the escape of stored air in micro-/nano-pits led to large increase in dielectric constant in parallel plate sensors. To produce the porous structure of EIPG electrode, anodic aluminum oxide (AAO) template with the hole diameter of 200 nm and depth of 30 μm was utilized instead of the flat silicon substrate, as shown in Fig. 1c. Deposited carbon atoms were accumulated on AAO surface except for the hole-area, thus producing similar structures on the deposited EIPG layer (Figs. 1d, S2). Furthermore, the morphology and diameter of pits on EIPG layer could be controlled by the aspect ratio, which is potential to be applied in wearable devices with surface modification. Figure 1e illustrates the Raman spectrum of the EIPG electrode. The peaks in Raman shift curve at 1350, 1580, and 2700 cm^−1^ represent D band, G band, and 2D band, respectively. The ratio of the intensity of D-Raman peak and G-Raman peak (*I*_*D*_*/I*_*G*_) is often used for the characterization of benzene cluster size [34]. Thus, the graphene crystallite size can be calculated to be 1.85 nm according to *I*_*D*_*/I*_*G*_. Furthermore, the graphene sheets plan is perpendicular to the substrate according to our formal research. The size of graphene sheets crystallite is essential to electrical properties, which can be controlled by the electrons irradiation energy during the deposition process [35]. Besides, the EIPG-based devices exhibit outstanding mechanical stability and pressure tolerance under external stimuli, thus enabling sensors with high stretchability and reliability [30]. Figure 1f presents the plan-view of EIPG layer where EIPG sheets with various orientations are observed by high-resolution transmission electron microscope (HRTEM). Whereas the EIPG sheets with perpendicular orientation can be identified in cross section of the electrode in Fig. 1g.

**Fig. 2:**
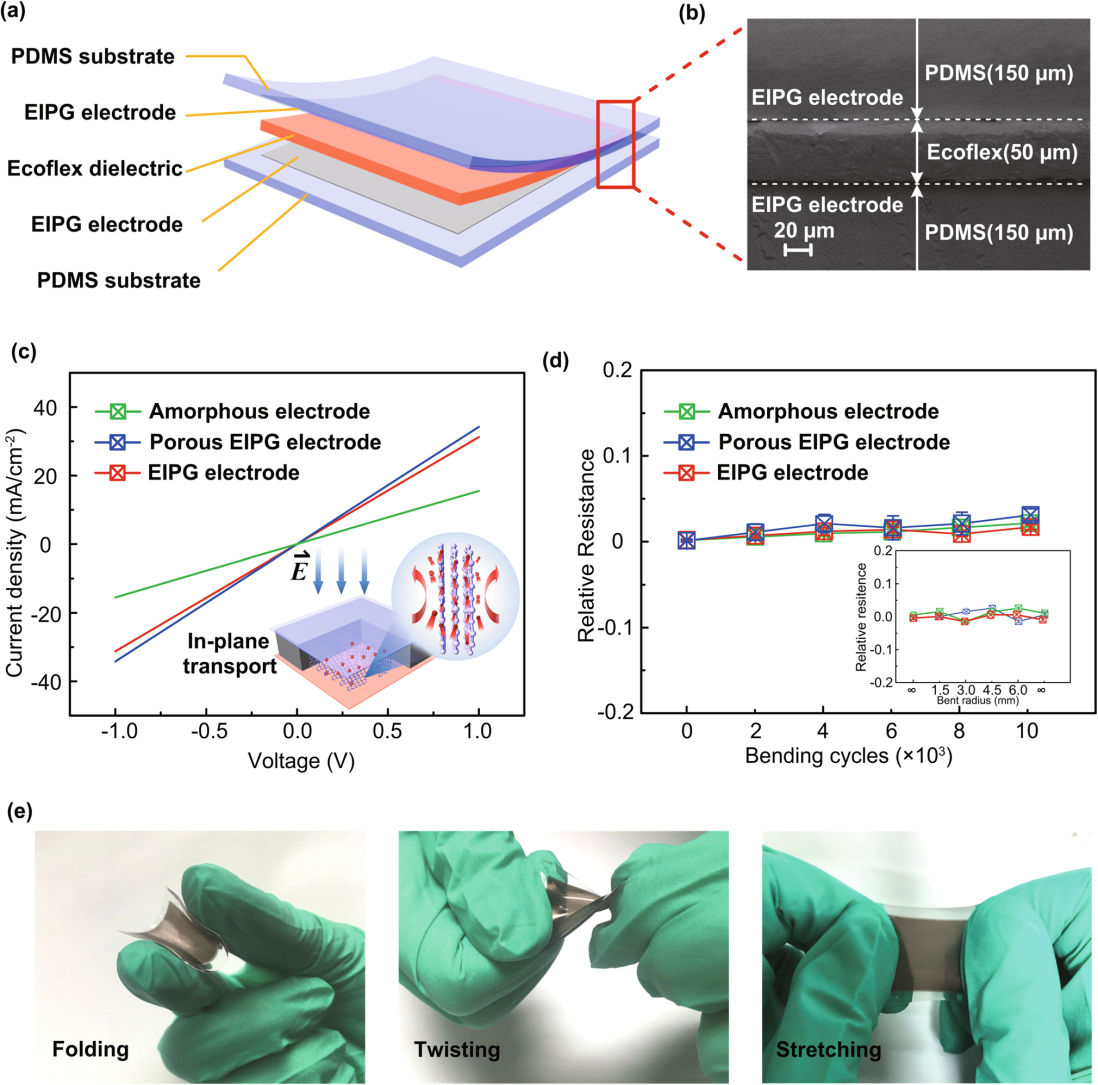
**a** Schematic view of the active layers of the EIPG composite-based touch sensor. **b** Cross section of the layer structure of EIPG electrodes. **c** Conductive properties as a function of various electrodes and the inset figure shows in-plane transportation of electrons in perpendicular graphene flakes. **d** Relative resistance versus changing bending cycles and in the inset figure versus bending radius. **e** Photographs of assembling sensor device (4 cm^2^) under folding, twisting and stretching

Figure 2a presents the schematic illustration of EIPG wearable touch sensor using hierarchically engineered electrodes and Ecoflex dielectric layer, which is low-cost, easy-fabricated, and environmentally degradable. Usually higher sensitivity of capacitance sensor can be achieved with dielectric elastomers of larger dielectric constant. Thus, polymers with large permittivity are often used as the dielectric layer to improve the sensitivity of the sensor [36], such as Ecoflex silicone elastomers, polyvinylpyrrolidone (PVP), and polyvinylidene fluoride (PVDF). As shown in Fig. 2b, the cross-sectional image of the assembled device clearly shows the interface between PDMS substrate and embedded Ecoflex dielectric with a thickness of ≈ 50 μm. Here, the electrical conductivity performance of EIPG electrode is maintained with porous structures, which is presented in Fig. 2c and the EIPG electrode exhibits less graphene-sheets-barrier with perpendicular graphene flakes to the current collector, providing electrons with in-plane transportation movements (inset Fig. 2c). Note that the intrinsically low conductivity of amorphous carbon film is much improved after EIPG flakes are formed. The in-plane conductivity is also a key factor for lateral conduction and large scale application. Here, the square resistance of EIPG electrode is measured to be 0.29 kΩ sq^−1^ based on four-probe experiments, which is comparable to square resistance (0.20 kΩ sq^−1^) of the single-walled carbon nanotube networks [37]. Besides, the mechanical property is essential for flexible devices [38]. Figure 2d shows the stability of relative resistance (Δ*R/R*) of each electrode after bending 10,000 cycles at a bending radius of 6 mm and the inset Fig. 2d presents the relative resistance measured at different bending radii, indicating high reliability during repeating deformation process. The maximum variations of relative resistance are only 4%, which can guarantee the reliability even at the bending radius of 6 mm. Furthermore, they perfectly recover after returning to an unbent state during repeated cycles, which ensures the promising application in flexible and wearable capacitance electrodes. The porous EIPG-based touch sensor exhibits mechanical robustness under various mechanical deformations such as folding, twisting, and even stretching conditions, as shown in Fig. 2e. Furthermore, in most previous works, the dielectric layer was fabricated in a separate process and then combined with capacitance electrodes together using a simple contact method. In contrast, here Ecoflex is spin-coated on the EIPG electrode followed by solidification and assembling, which prevents the peeling-off or delamination of flexible electrodes from elastomers, leading to the high reliability of the sensors.

### Pressure-Sensing Capability of Device

**Fig. 3:**
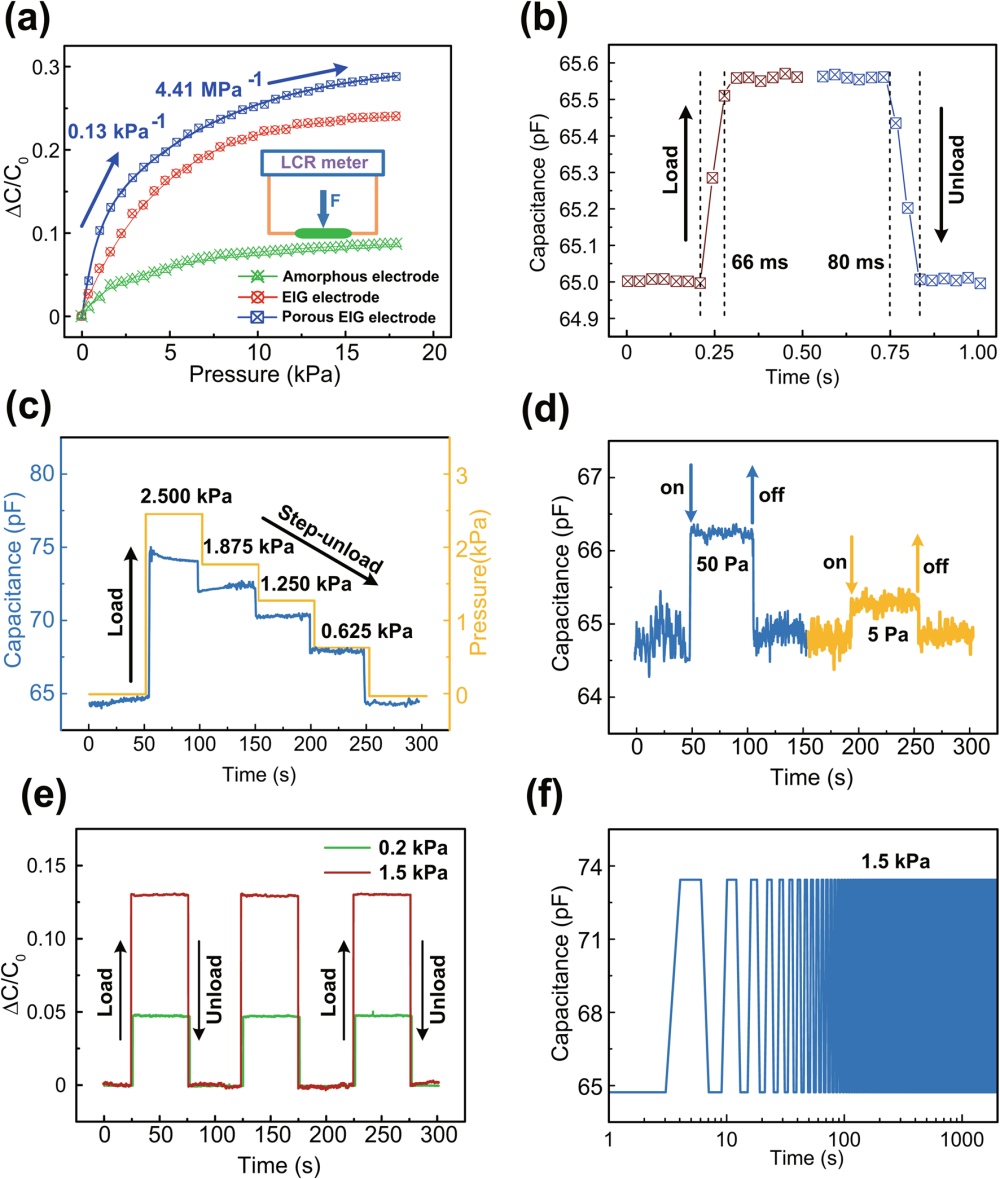
Pressure-sensing performance of the EIPG touch sensor. **a** Relative capacitance (Δ*C*/*C*) versus normal pressure with various electrode types. **b** The instant response of the EIPG touch sensor shows response times of 66 ms. **c** Transient response to the load and step-unload process. **d** Transient response of the sensor when placing and removing a paper or a polytetrafluoroethylene sheet on the substrate, corresponding to a pressure of only 50 and 5 Pa, respectively. **e** Δ*C*/*C* versus time under applied pressure of three cycles. **f** Durability testing under external pressure of 1.5 kPa

The pressure-sensing capability of the EIPG composites sensor was performed using an LCR (inductance, capacitance, and resistance) meter when applying an external force to the device. Figure 3a illustrates the capacitance change ratio (Δ*C/C*) along with the increasing applied normal pressure in terms of sensors based on conventional amorphous carbon film electrode, EIPG electrode, and porous EIPG electrode, respectively. Moreover, the relative capacitance (Δ*C/C*) presents a quasi-bilinear relationship with external pressure at each regime (low-pressure regime of ≤ 0.1 kPa and high-pressure regime of ≥ 10 kPa), which is caused by the incompressive properties of Ecoflex dielectric layer. Pressure sensitivity is defined as the slope of the trace to evaluate the performance of capacitance sensor. Here, for low- and high-pressure regime, the sensitivity of porous EIPG sensor is 0.13 kPa^−1^ and 4.41 MPa^−1^, respectively. The EIPG capacitance shows higher sensitivity to amorphous carbon capacitance at low-pressure regime, originating from the more efficient electron transportation process induced by EIPG flakes among carbon film, and the sensitivity of porous EIPG capacitance is further enhanced compared to flat EIPG sensor, which arises from hierarchical EIPG nanostructure in the layer interface. The concentration of EIPG can be increased by applying higher electron irradiation energy during depositing process. According to our previous research, the electrical properties of EIPG layer would exhibit relatively good performance under the irradiation voltage of 100 V [39]. This is the reason for preparing EIPG electrode under irradiation voltage of 100 V in our work. Moreover, the detection of the limit of flexible capacitive pressure sensors reported in the previous studies is summarized [40–42], making our device exhibit relative superior performance (Table 1). The dielectric constant and mechanical properties of dielectric layer are also essential factors for comprehensive performance of sensors. Higher sensitivity might be obtained if we use porous Ecoflex dielectric layer, but the detecting range would be much reduced. Our device with dielectric layer thickness of only 50 μm exhibits relatively high sensitivity and fast response time under wide pressure range, presenting superior integrate performance of EIPG materials.

**Table 1:** Summary of sensitivity and testing range of flexible sensor devices

Device type	Materials	Sensitivity	Testing range	Response time	References
Capacitance	EIPG/Ecoflex	0.13 kPa^−1^	5 Pa–20 kPa	66 ms	This work
Capacitance	CNT/Ecoflex	0.05 kPa^−1^	0.75 Pa–25 kPa	63 ms	[12]
Capacitance	Fiber/Silicone	0.0121 kPa^−1^	0–100 kPa	–	[16]
Capacitance	AgNWs/PVDF	2.94 kPa^−1^	3–7500 Pa	< 50 ms	[21]
Capacitance	Fiber/PDMS	0.27 kPa^−1^	38 Pa–55 kPa	~ 340 ms	[22]
Capacitance	AgNWs/ATG Tape/Pu film	3.3 MPa^−1^	1 kPa–1 MPa	–	[23]
Resistance	Carbon film/PDMS	1071-GF	0–16% strain	–	[30]
Capacitance	AgNWs/Ecoflex	0.15 kPa^−1^	2–7 kPa	200 ms	[41]
Resistance	Graphene-based Fiber	0.82-GF	0–200% strain	< 100 ms	[42]

Figure 3b illustrates the sensor exhibits rapid response time of only 66 ms, which is comparable to conventional types of flexible pressure sensors. Furthermore, the sensor device shows highly reversible capacitive response. As shown in Fig. 3c, while the pressure of 2.5 kPa is loaded to the sensor and step-decreased, the capacitance value response corresponding to the external pressure trend. Moreover, Fig. 3d presents the transient response of the touch sensor while placing and removing small objects such as a paper and polytetrafluoroethylene sheet on the sensor, which refer to quite low pressure of only 50 and 5 Pa, respectively. In fact, we also observed the super high sensitive performance while measuring the sensing ability when blowing air gently on the sensor (Fig. S3), indicating potentials in detecting much lower pressure applications. As shown in Fig. 3e, the device can also operate stably under continuous force loading/unloading cycles. Besides, Fig. 3f illustrates excellent reliability of our sensor by testing the capacitance while repeatedly loading and unloading an external pressure of 1.5 kPa for over 10,000 s. More importantly, the device can also maintain good performance under various long-time loading conditions (Fig. S4).

### Application of Touch Sensor

**Fig. 4:**
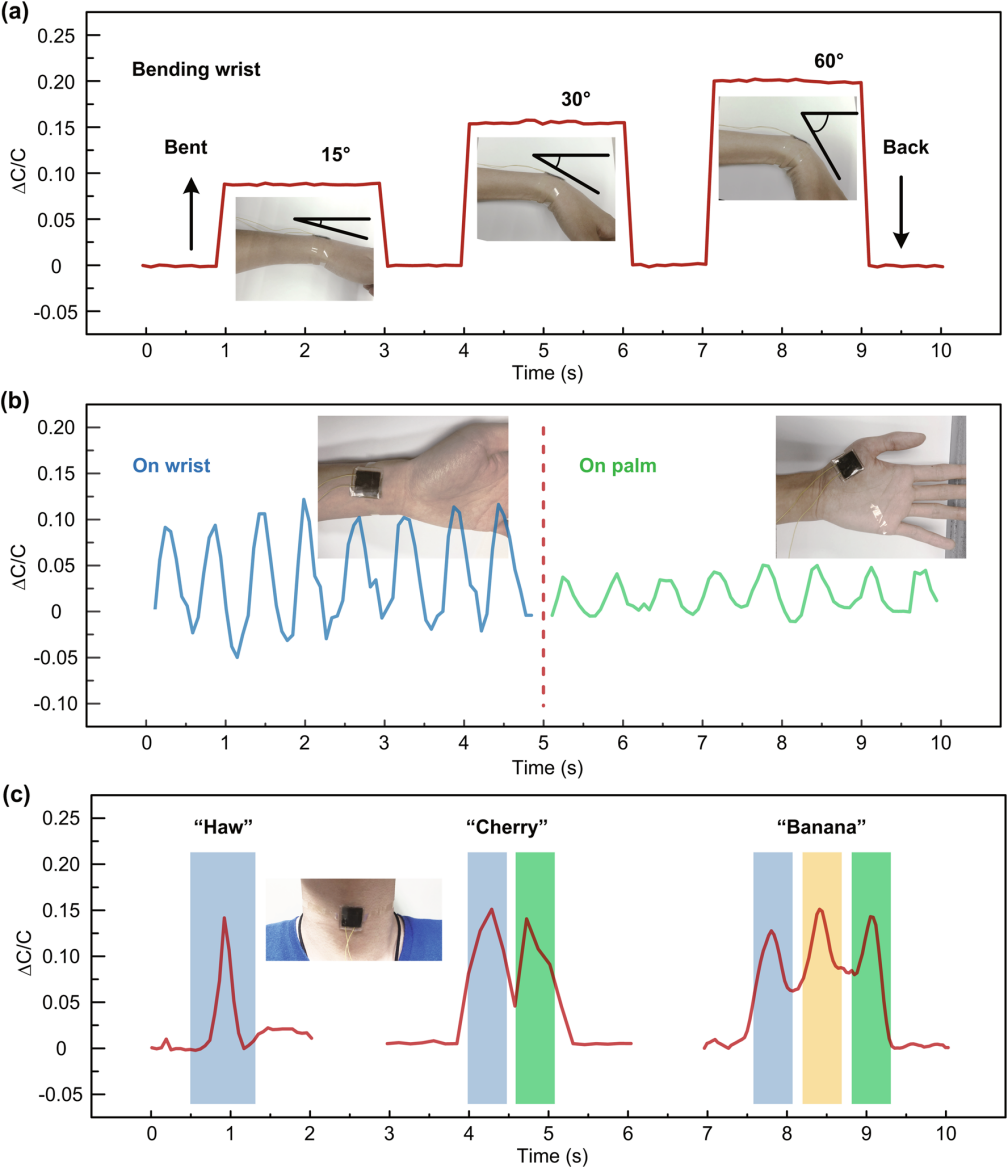
Application of the wearable sensor. **a** Capacitance response plot obtained from the sensor when wrist bents with different angles. **b** Capacitance response of the sensor on the bloodstream in the wrist and the palm. **c** Transient response of the flexible sensor to speaking vibrations under the pronunciation of “haw,” “cherry,” and “banana”

Besides good pressure-sensing capability, our device was explored to discern human motions, which is strongly desired for prosthetics and collecting clinical information. Figure 4a shows the application of our touch sensor as a wearable device fixed onto the human wrist. The sensitivity of sensor to writs bending angles was analyzed by measuring the capacitance under specific angles of 15°, 30°, and 60°, step by step, and the wrist was held for 2 s at each angle, respectively. The relative capacitance increased along with the bending angle of wrist from 15° to 60°, due to the compression induced by the bending process. Whereas the relative capacitance would return back to zero while unbending the wrist due to the release of the compressive strain. The sensor can rapidly respond to angle change and remain constant at specific angles, indicating great potentials in detecting movements of robotic joint. Figure 4b illustrates the real-time measurement of wrist pulse (~ 96 beats min^−1^) detected by our device to collect clinical information, where the external beat pulse would apply vibration force to the PDMS substrate of sensor, thus the change of dielectric layer thickness induces the fluctuation of capacitive value, which reflects the wrist pulse of human body. To check whether the sensor could distinguish pulses of different body parts, the sensor signal collecting from palm pulse is also plotted, where there are also pulse beats induced by bloodstream. Later, the device was attached onto the volunteer neck to monitor the throat vibrations during pronunciation. The relative capacitance vibrations versus time are illustrated in Fig. 4c. As the curves show, one, two, and three peaks are observed when volunteer pronounces a monosyllabic word as “haw”, disyllabic word as “cherry” and trisyllabic word as “banana,” respectively, which indicates the excellent detection capability of flexible sensor to speaking. Besides, more testing results about the stability of detecting the voice are summarized in Fig. S5. Based on the efficient recognition to voice, this flexible sensor device is attractive for the people with damaged vocal cords. These results show that our sensors provide vast potentials for applications in robotic e-skins, monitoring human clinical information, and prosthetics.

### Dual Parameter of Touch Sensor

**Fig. 5:**
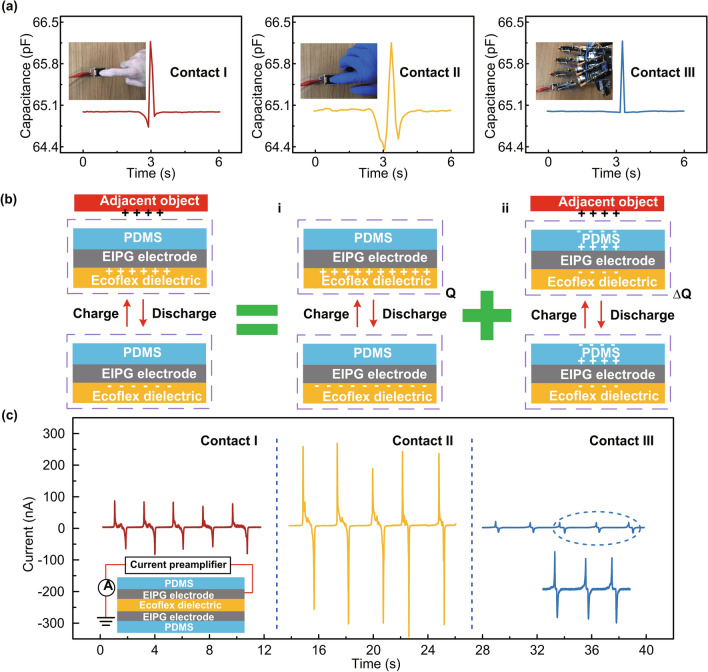
a Capacitance change under external pressure applied (slowly approach, touch and leave) by bare hand (contact I), with nitrile rubber on (contact II) and metallic mechanical hand (contact III), respectively. **b** Mechanism for capacitance decrease when adjacent object approaches (i) charges distribution on capacitance without adjacent object (ii) fixed charges induced by adjacent objects. **c** Dual-parameter function according to short-circuit current generated by the single-electrode connection method

We observed an interesting phenomenon that capacitance would decrease when a finger touched the device, which was also reported in some previous works about capacitive sensors [43]. By contrast, the piezoresistance sensor shows no similar phenomenon according to the testing curve [44–48]. After further investigating this process, we realized that the decreased value of capacitance was closely related to the nature of the adjacent materials. Figure 5a illustrates the capacitance curve under external pressure (slowly approach, touch and leave) applied by bare hand (contact I), with nitrile rubber on ((contact II) and metallic mechanical hand (contact III), respectively. Note that the capacitance value decreases even before adjacent objects contact with the sensor in contact I and II due to the disturbance of the fringing electric field before the dimension change of the dielectric layer. While the curve of contact III shows nearly no decrease when a metallic finger approaches due to the electrostatic shielding process of metal. Before touching the sensor, the adjacent objects like our hands might carry charges due to the human body capacitance. These charges would reduce the value of sensor capacitance in contact I and contact II. While the metal objects would carry much less charges due to the electrostatic shield of metal, thus the sensor capacitance would stay still before the external pressure is applied in contact III. After the adjacent objects have touched the sensor, the capacitance value would change due to the variation of dielectric layer thickness under applied stimuli. During this process, the triboelectric charges induced by the adjacent objects and the PDMS substrate might also change the capacitance value. However, the external pressure would give out far more contributions to the change of capacitance value than the triboelectric charges. As shown in Fig. 5b, the capacitance of this sensor during the measuring process can be defined as *C* = *Q/U*, where *Q* is the quantity of induced electric charges and the *U* is the applied voltage between two electrodes (alternating voltage of 1 V in this work). The induced charges distribution would change due to the potential difference between an adjacent object and sensor electrode. Comparing with the charges distribution of capacitance without adjacent objects [Fig. 5b(i)], part of the induction charges under applied voltage is fixed at the electrode [Fig. 5b(ii)], which reduces the quantity of effective flowing electric charges. As the principle of superposition for the electric field, the updated capacitance of this sensor should be *C* = (*Q* − Δ*Q*)*/U*, where Δ*Q* is the quantity of fixed electric charges induced by the adjacent object. Moreover, according to the Gauss’s theorem (under uniform electric field assumption), Δ*Q* is determined by the potential difference between the electrode (*V*_1_) and the adjacent object (*V*_2_) as (*V*_1_ − *V*_2_)*/D* = Δ*Q/ε*_0_, where *D* is the distance between electrode and adjacent object and ε_0_ is the vacuum permittivity. Therefore, the final equivalent capacitance of this sensor should be *C* = (*Q* − *ε*_0_ (*V*_1_ − *V*_2_)*/D*)*/U*. Note that, when the finger comes close to the sensor, *D* would decrease and *C* would drop rapidly according to the negative correlation relationship, corresponding to the capacitance curve when approaching and leaving (Fig. S6).

To gain insight into the dual-parameter detecting function of our touch sensor, short-circuit current generated by connecting the EIPG electrode and ground when adjacent objects repeatedly touched the PDMS surface was analyzed (Fig. S7). As shown in Fig. 5c, the short-circuit of ~ 100, ~ 300, and ~ 10 nA is generated when repeatedly touching and releasing the sensor by bare hand, with nitrile rubber on and metallic mechanical hand, respectively. The positive correlation between this short-circuit current and decreasing value of capacitance when a finger touches the sensor further confirms that, the potential difference between the sensor electrode and adjacent object causes the capacitance fluctuation when adjacent objects approach, indicating potential applications in internet of things security system to recognize bio-/abio-individuals.

## Conclusions

In conclusion, we have developed a highly sensitive, flexible, and dual-parameter touch sensor using a hierarchically engineered elastic dielectric layer and less graphene-sheets-barrier porous EIPG electrode. The EIPG touch sensor in this work is capable of simultaneously sensing various external subtle stimuli such as twist bending, bloodstream, voice vibration, and even adjacent object materials into one device. The sensitivity of our touch sensors with dielectric layer thickness of only 50 μm is 0.13 kPa^−1^ and 4.41 MPa^−1^ for pressure below 0.1 kPa and above 10 kPa, respectively, and fast response time of ~ 66 ms under even quite low pressure of 5 Pa. Moreover, our sensor device could recognize the adjacent object using the single-connection method, according to the decreasing value of capacitance when approaching and leaving. This structural design of a flexible touch sensor, combine the advantages of EIPG electrode and porous surface modification, is a promising strategy for assembling graphene-based sensor devices and surface modification of carbon materials.

## Electronic supplementary material

Below is the link to the electronic supplementary material.

Supplementary material 1 (PDF 593 kb)
