# Supplementary material for: Electron-Induced Perpendicular Graphene Sheets Embedded Porous Carbon Film for Flexible Touch Sensors
Source: Nanomicro Lett. 2020 Jun 25;12:136. doi: 10.1007/s40820-020-00480-8 (PMC7770710; doi:10.1007/s40820-020-00480-8)
Supplement: Supplementary file 1 — Supplementary material 1 (PDF 593 kb) [file 40820_2020_480_MOESM1_ESM.pdf]

Supporting Information for

Electron-Induced Perpendicular Graphene Sheets Embedded Porous Carbon Film for Flexible Touch Sensors

Sicheng Chen[1], Yunfei Wang[1], Lei Yang[1,*], Fouad Karouta[2], Kun Sun[1]

[1]Key Laboratory of Education Ministry for Modern Design and Rotor-Bearing System, Xi'an Jiaotong University, Xi'an 710049, People's Republic of China

[2]Research School of Physics, The Australian National University, Canberra, 2601 ACT Australia

*Corresponding author. E-mail: yanglxjtu@xjtu.edu.cn (Lei Yang)

Supplementary Figures

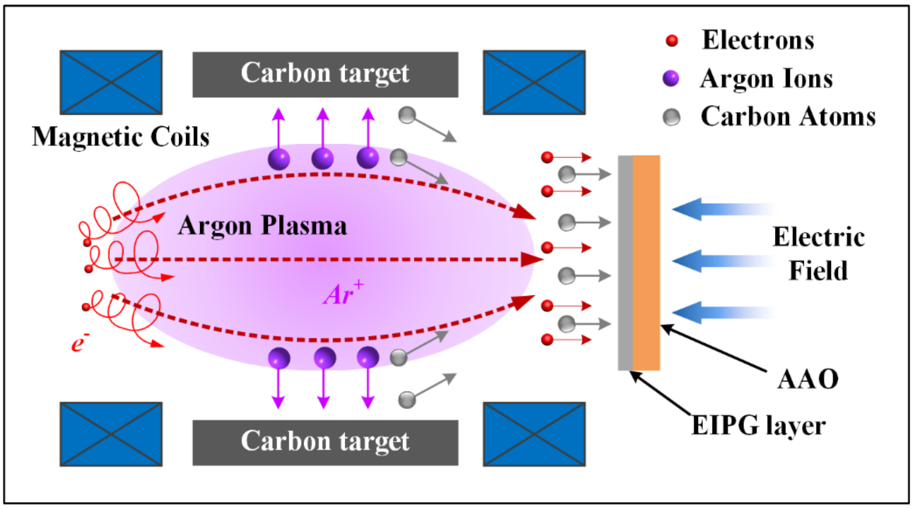

Fig. S1 Schematic illustrations of the ECR sputtering system

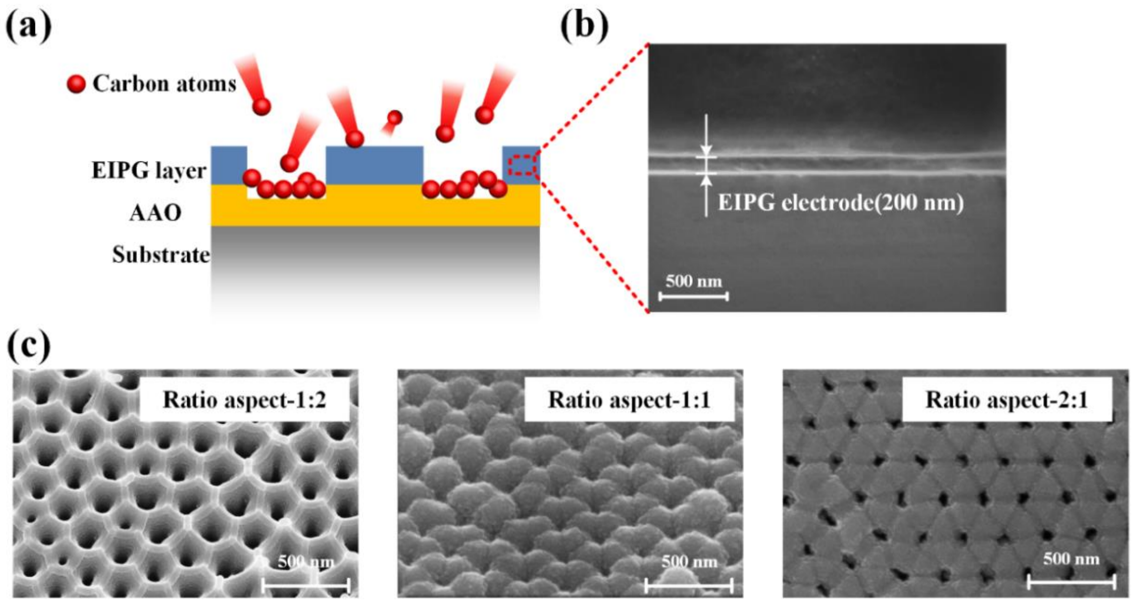

Fig. S2 a Schametics of accumulating carbon atoms and **b** Field-emission scanning electron micrographs of EIPG cross-section geometry and **c** Morphology of EIG layers under different ratio aspect (Ratio aspect=pit-depth/pit-diameter)

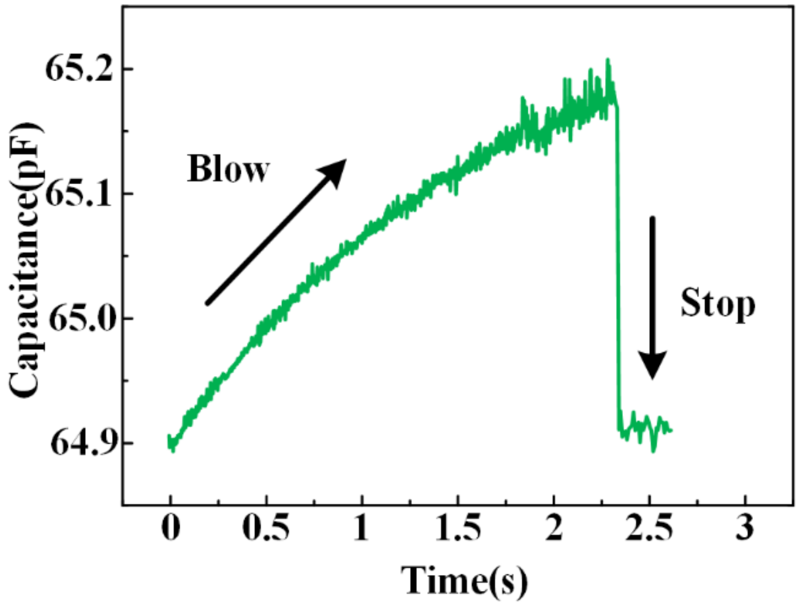

Fig. S3 Capacitance change when blowing on the sensor

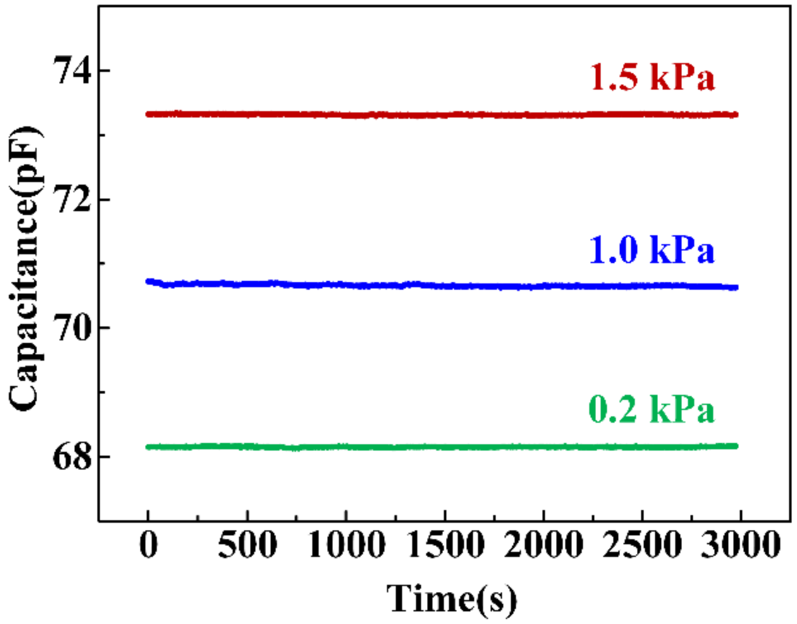

Fig. S4 Durability of capacitance under long-time loading process

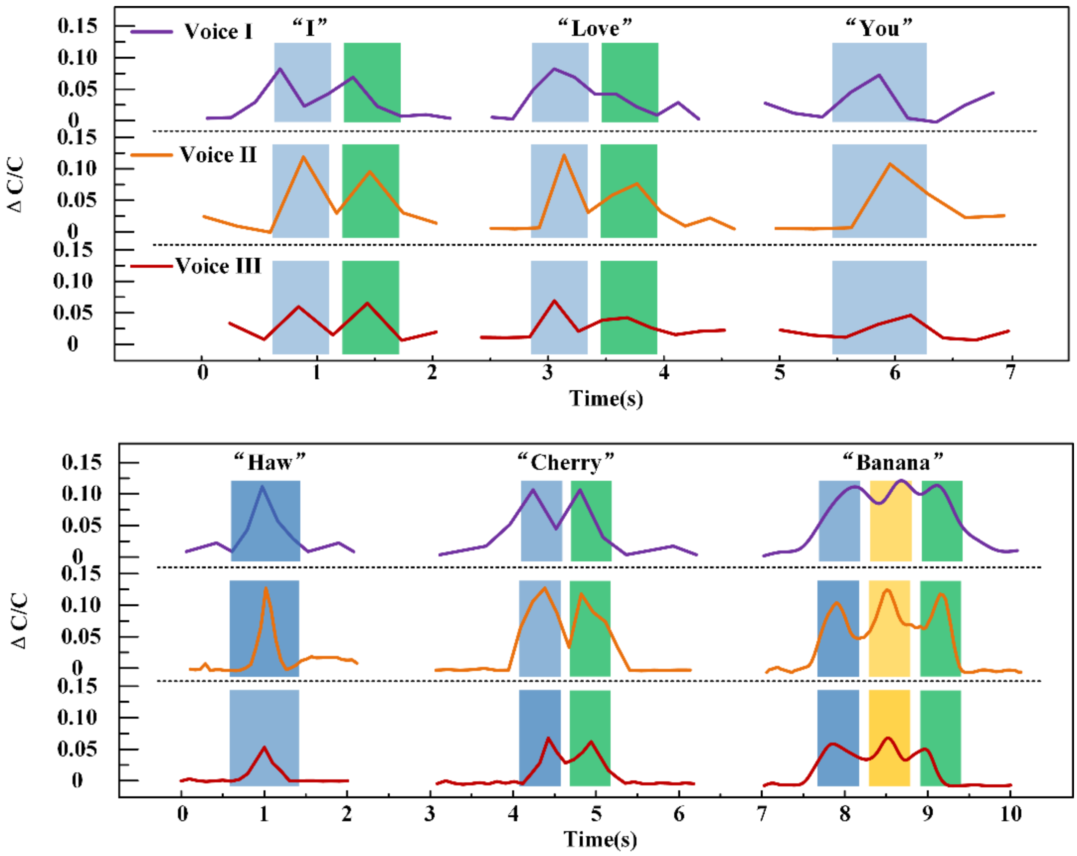

Fig. S5 Sound detection application of the EIPG sensor

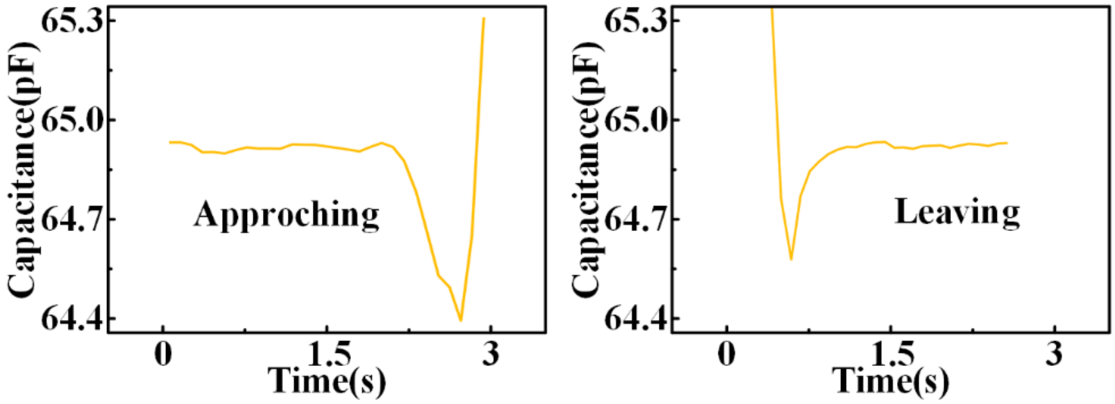

Fig. S6 Capacitance changes when approaching and leaving (Capacitance decreased rapidly when adjacent objects approach, like hyperbolic curve as the element of "1/D", the turning point of leaving is slightly higher than that of approaching for the charge exchange between capacitance surface and adjacent object)

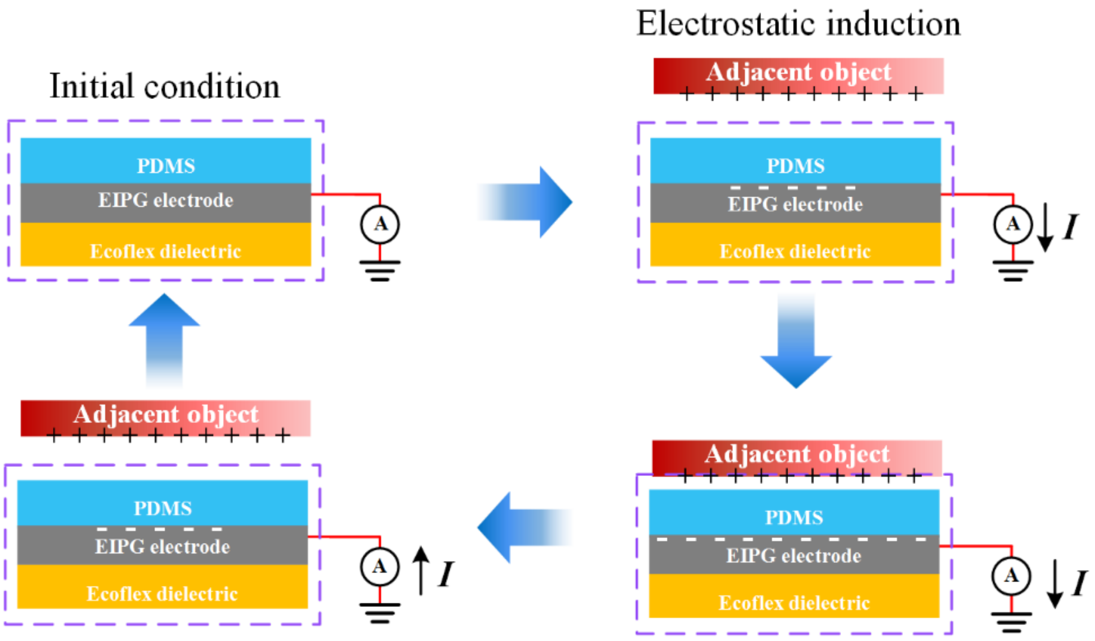

Fig. S7 Schematic illustrations for the working process of bimodal function when detecting adjacent objects using single-electrode connection
